# Using Vital Dyes to Trace Uptake of dsRNA by Green Peach Aphid Allows Effective Assessment of Target Gene Knockdown

**DOI:** 10.3390/ijms18010080

**Published:** 2017-01-03

**Authors:** Vineeta Bilgi, John Fosu-Nyarko, Michael G. K. Jones

**Affiliations:** Plant Biotechnology Research Group, Western Australia State Agricultural Biotechnology Centre, School of Veterinary and Life Sciences, Murdoch University, Perth, WA 6150, Australia; vineetabilgi@gmail.com

**Keywords:** RNA interference (RNAi), double stranded RNA (dsRNA), green peach aphid, *Myzus persicae*, vacuolar (H^+^)-ATPase (*vha-8*), vital dyes, neutral red, acridine orange

## Abstract

RNA interference (RNAi) is an effective tool to study gene function. For in vitro studies of RNAi in insects, microinjection of double-stranded (ds)RNA may cause stress. Non-persuasive oral delivery of dsRNA to trigger RNAi is a better mode of delivery for delicate insects such as aphids because it mimics natural feeding. However, when insects feed ad libitum, some individuals may not feed. For accurate measurement of gene knockdown, analysis should only include insects that have ingested dsRNA. The suitability of eleven dyes was assessed to trace ingestion of dsRNA in an artificial feeding system for green peach aphids (GPA, *Myzus persicae*). Non-toxic levels of neutral red and acridine orange were suitable tracers: they were visible in the stylet and gut after feeding for 24 h, and may also attract aphids to feed. Nymphs stained with neutral red (0.02%) were analysed for target gene expression after feeding on sucrose with dsRNA (V-ATPase, *vha-8*). There was a greater reduction in *vha-8* expression and reproduction compared to nymphs fed the diet without dye. The results confirm the importance of identifying aphids that have ingested dsRNA, and also provide evidence that the *vha-8* gene is a potential target for control of GPAs.

## 1. Introduction

Insect pests cause direct and indirect damage to crops and are responsible for millions of dollars of losses in crop production worldwide [1]. Aphids (Hemiptera: Aphididae) are the largest group of insects that predominantly feed on plant phloem sap. Heavily infested plants are usually stunted, weak, and have reduced photosynthesis that can result in reduced crop yields. Of the 4700 aphid species described to date, more than 190 have been proven to transmit plant viruses to most of the world’s important crops [1,2]. The green peach aphid (GPA), *Myzus persicae* (Sulzer), is a polyphagous insect which feeds on plants from over 40 different families including Brassicaceae and Solanaceae and is also a vector for more than 100 viruses [1,3]. These characteristics, combined with its cyclical parthenogenesis, telescoping of generations and recent outbreaks of resistance to chemical insecticides, make GPA a successful pest [4,5,6]. This makes it imperative to look for other strategies for its control, which may also be more sustainable and environmentally friendly. RNA interference (RNAi) is used widely as a tool to study gene function and is being explored as an alternative strategy for crop protection against several different plant pathogens and pests including nematodes and some insects [7,8,9,10].

RNAi is a natural mechanism of gene regulation in eukaryotes. It can be triggered by the introduction of double-stranded RNA (dsRNA) into an organism leading to sequence-specific suppression of homologous mRNA and loss-of-function of its protein [11,12,13]. It is now an invaluable tool for studying gene function in many organisms [9,10,14,15]. Several approaches have been used to deliver dsRNA to insects or tissues, such as soaking cell-lines, microinjection, and oral feeding of artificial diets and via transgenic plants [7,16,17,18,19,20]. In many cases of in vitro RNAi studies in insects, dsRNA has been delivered by microinjection or orally, in which insects are allowed to feed ad libitum [21,22,23,24,25]. Microinjection of long dsRNA or small interfering RNA (siRNA) into the abdomen or thorax involves the use of very fine glass needles to administer a known amount of dsRNA into various nymphal stages of insects such as *Acyrthosiphon pisum*, *Bemisia tabaci*, and *Nilaparvata lugens* [26,27,28,29]. This method requires skill to reduce undue stress, injury or death, and requires careful selection of appropriate size of needles as well as empirical injection volumes and doses of dsRNA [27]. Microinjection can be laborious and may not be suitable for large-scale functional analyses of target genes, and for small nymphs of some insects the procedure may cause substantial injury and stress [24,25,30,31].

Oral delivery of dsRNA to insects in vitro (also referred to as artificial feeding) is a convenient, non-invasive alternative, and allows the use of delicate nymphs, and ad libitum feeding, which is closer to the natural feeding behaviour of aphids [24,25,31]. Artificial feeding is also well-suited for large scale functional analyses and has been used successfully for example, to screen 290 dsRNA targets in *Diabrotica virgifera virgifera* [7]. However, one of the limitations of oral delivery is that it is difficult to determine the amount of dsRNA ingested by the insect or to assess if any solution is taken up at all. Most published studies on in vitro RNAi involving artificial feeding of insects do not use markers to trace uptake of solutions. In such experiments it is assumed that all insects have ingested RNAi triggers, and the measure of gene silencing is averaged [16,19,32,33,34]. This is likely to result in underestimation of gene knockdown if in fact not all insects have ingested dsRNA. However, if uptake of solution can be traced and confirmed, those insects that have taken up artificial feed containing dsRNA can be identified, and only those used to deliver a more accurate analysis of gene silencing. Fluorescent-labelled Cy-3^®^ has been used to investigate uptake of dsRNA in Hemipterans such as the glassy-winged sharpshooter, *Homalodisca vitripennis*, the grain aphid, *Sitobion avenae*, and the potato/tomato psyllid *Bactericera cockerelli* [35,36,37]. However, Cy-3^®^ labelling is expensive when undertaking replicated in vitro RNAi experiments involving many different target dsRNAs/genes. For such large-scale experimentation the use of inexpensive dyes to trace uptake of dsRNA would allow effective identification of those aphids that have fed.

In this report, we assessed the suitability of eleven inexpensive dyes to trace the uptake of dsRNA by GPAs, their effect on integrity of dsRNA and on the behaviour, activity and reproduction of the aphids. Ingestion of low concentrations of two vital dyes, neutral red and acridine orange, was evident as the dyes were visible in the aphid body and did not affect the behaviour of GPA or the stability of dsRNA. We also show that when neutral red-stained aphids were analysed after ingesting dsRNA of the vacuolar (H^+^)-ATPase subunit E-like (*vha-8*) gene, there was a greater reduction in gene expression and reproduction than when aphids were fed a diet of dsRNA without the dye.

## 2. Results and Discussion

### 2.1. Identification of Appropriate Dyes to Trace Uptake in Aphids

“Artificial diets” for in vitro RNAi studies with insects normally comprise 30% sucrose, known amounts of dsRNA or siRNA with or without chemical agents such as lipofectin. In this study, the basic artificial diet was 30% sucrose to which dsRNA and/or dyes were added either to study gene knockdown or the potential of the dyes as markers to trace ingestion of solutions by GPAs. Important criteria for selecting these dyes were that an appropriate dye should be taken up orally by aphids without any adverse effect on behaviour, growth, development and survival or on the integrity of dsRNA, that the dye can be visualised with or without a microscope and should not react with sucrose to produce toxic by-products over the period of the experiment. Based on these criteria eleven inexpensive dyes were tested for their suitability in the study: these were two fluorescent dyes: fluorescein isothiocyanate (FITC; Sigma Aldrich 46952, Castle Hill, NSW, Australia), and fluorescein diacetate (FDA; Sigma Aldrich F7378, Castle Hill, NSW, Australia), six vital dyes: phloxine B (PB; Sigma Aldrich P2759, Castle Hill, NSW, Australia), neutral red (NR; VWR International, Brisbane, QLD, Australia), congo red (CR; VWR International, Brisbane, QLD, Australia), methylene blue (MB; VWR International, Brisbane, QLD, Australia), acridine orange (AO; VWR International, Brisbane, QLD, Australia) and fast green (FG; VWR International, Brisbane, QLD, Australia), one non-vital dye, acid fuchsin (Sigma Aldrich F8129, Castle Hill, NSW, Australia), and two food colours: Red (RFC) and Yellow (YFC) (Queen Fine Foods, Alderley, QLD, Australia).

**Figure 1:**
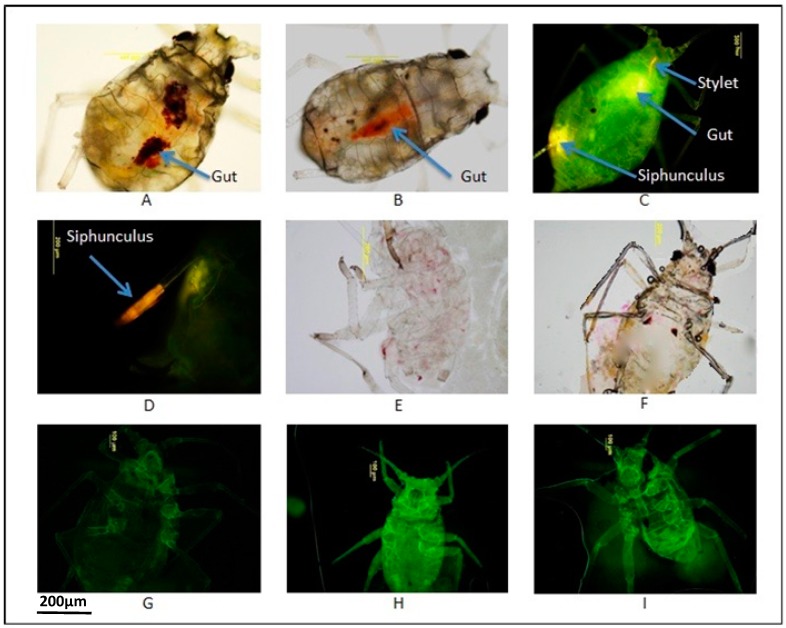
Visualisation of neutral red, congo red, acridine orange, phloxine B, fluorescein isothiocyanate, and fluorescein diacetate in the bodies of GPAs after feeding for 24 h. (**A**) Presence of CR in the gut; (**B**) Presence of NR in the gut; (**C**,**D**) Presence of AO in the stylet, gut and siphunculus; (**E**,**F**) Presence of PB in moult and in the abdomen respectively; (**G**–**I**) Autofluorescence in GPA fed with sucrose, FITC and FDA respectively.

In a preliminary study to identify which of the dyes could be ingested and were observable in the aphid body without detrimental effects on growth and development, three concentrations each of the fluorescein dyes; FITC at 0.5, 1, and 2 mg/mL and FDA at 0.01%, 0.001%, and 0.0001% and one of each of the other dyes; PB at 0.75%, NR (0.5%), CR (0.5%), AF (0.25%), MB (0.5%), FG (0.25%), AO (0.05%), RFC (1:10) and YFC (1:10) were each mixed with 30% sucrose and provided in 5 mL yellow cap tubes to 20 nymphs to enable them to feed for 24 h. After this period, the aphids were observed with an Olympus BX-51 microscope under FITC filter or UV filter for FITC, FDA, AO and a compound microscope for all other dyes. The dyes were visualised in six aphids fed with AO, five fed with NR and in only two and one respectively fed with CR and PB. In these aphids the dyes were observed in the stylets and gut, and for CR and NR, appears to accumulate in the gut as a relatively large dense colouration were visible without the aid of a microscope (Figure 1). For AO-fed aphids, similar intensities of fluorescence were observed in the stylet and guts whereas for some, additional fluorescence resulting from AO ingestion was observed in the siphunculi (Figure 1). In the only aphid that could be seen to have ingested PB, the dye was faintly visible in the moult, distributed throughout the body, especially in the abdomen in dotted pattern (Figure 1). Using an FITC filter at an excitation wavelength of 490 nm, autofluorescence was observed in aphids presented with 30% sucrose alone and the intensity could not be differentiated readily from fluorescence possibly emitted by aphids after ingesting different concentrations of FITC and FDA (Table 1). In other insects, for example screwworm, FITC does not appear to impart a strong enough signal to living tissues and is not considered a good marker [38]. No color was detected in aphids presented with the two food colours (RFC and YFC) and the dyes AF, MB, and FG. This may be because the concentrations of these dyes in the aphid body could have been too low to be visualised. Alternatively, the aphids may not have ingested the feed with these dyes perhaps because of properties of the dyes, such as colour intensity, surface tension, odour or taste. Like most insects, aphids rely on well-developed chemosensory and olfactory systems to detect semiochemicals in the environment and any undesirable component of these dyes could disrupt feeding leading to possible starvation and inactivity and/or death: this was similar to the observation made for GPA 24 h after feeding on the dyes [39]. For this reason, the concentrations could not be increased for further experimentation because such increase could have been more detrimental to survival or activity of the aphids (Table 1). However, for CR, NR and AO only a small percentage of aphids were inactive.

### 2.2. Optimisation of Non-Toxic Concentrations of AO and NR Visible in Aphid Bodies

**Figure 2:**
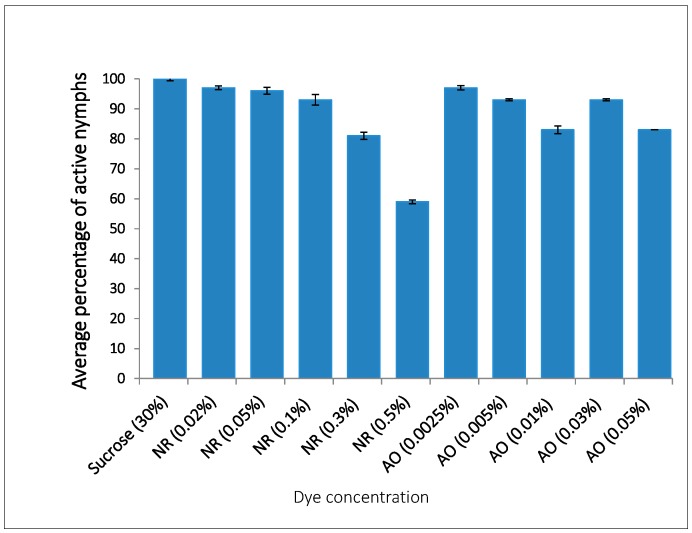
Percentage of active aphids after 24 h feeding on NR and AO suspended in 30% sucrose. Data represent three replicates with *n* = 10 nymphs per replicate.

Based on the number of aphids that survived after feeding for 24 h and also showed the presence of dye in the stylet, gut and siphunculi, five concentrations of NR and AO were selected for further investigation: the aim was to identify the lowest concentration that could be visualised in aphid bodies when ingested with little or no obvious effect on activity and feeding. Aphid activity was defined in this context and throughout the manuscript as significant response to external stimuli (e.g., touch with a paint brush) reflected in movement including but not restricted to change in direction of movement or a jolt or twitch or raising or lowering of the antennae within one minute of application of the stimulus—aphids which did not exhibit such activity were classified as inactive. This study was done because it was important that the appropriate concentration of dye, duration of exposure and stage of development of aphids be empirically determined before the dye was selected for inclusion in an artificial feed with dsRNA. Three replicates of 10 aphids, which fed from feeding sachets were analysed for each concentration. After 24 h, the nymphs had taken up feed with all concentrations of the dyes as evidenced by the stains in the bodies (Table 2). For both dyes, the highest numbers of live and active nymphs that showed the presence of dye was recorded for the lowest concentrations i.e., 0.0025% for AO and 0.02% for NR (Table 2), an indication that higher concentrations of the dyes may affect activity of the aphids. However, there were significantly more active aphids when exposed to the lowest concentrations of both dyes (*p* < 0.05) (Table 2 and Figure 2). The results indicate that at these concentrations, the dyes were not toxic to the aphids at least during the 24 h following ingestion as the number alive and their activity were not significantly different from those fed 30% sucrose alone. It was observed that the nymphs were attracted to sucrose because more ingested the feed of 30% sucrose with dyes and had visible dye in body than was the case for aphids exposed to feed with dye present in water (Table 2 and Figure 2).

For both dyes, the intensities observed in aphid bodies varied and there was no correlation between the concentrations of any of the dyes and the intensities in the body: these differences are probably a reflection of the random feeding behavior of aphids resulting in ingestion of different amounts of the dyes rather than a property of the dyes. However, the ability to visualise these dyes at concentrations that do not affect the phenotype of the aphids make these dyes attractive for research as demonstrated in this study. These observations reinforce both AO and NR as vital dyes which normally are stains/dyes that are easily or actively taken up by living cells/tissues at concentrations that do not cause harm or can be physiologically processed or pumped out of living cells without obvious deleterious effects. This is further supported by the use of AO as a fluorescence marker for insect tissues at concentrations of 0.001, 0.01, 0.1, 1, 2 and 5 mg/mL without any deleterious effect on growth, development, longevity, mating or oviposition of insects species of the order Diptera and Hymenoptera [38,40,41]. Similarly, the lower concentrations of NR used in this study were appropriate as >0.1% has been reported to be detrimental to GPA [42].

### 2.3. Double-Stranded RNA Quality Is Not Affected by Neutral Red or Acridine Orange

**Figure 3:**
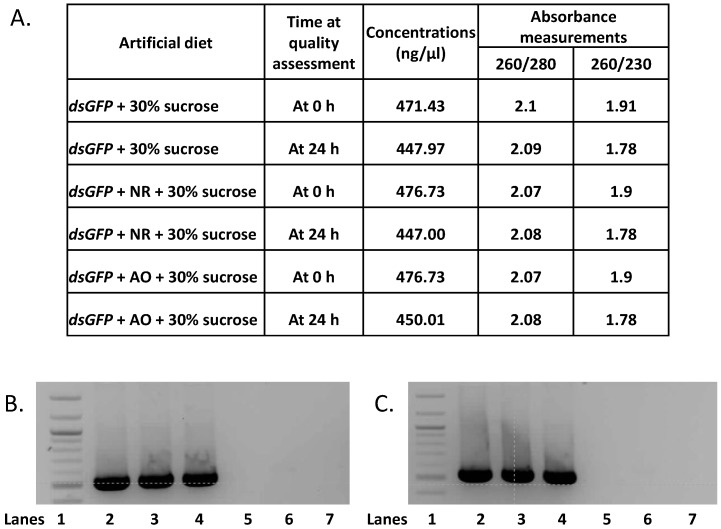
Quality of dsGFP mixed with dye and 30% sucrose (**A**); Concentration and absorbance ratios of dsGFP with and without dyes at 0 and 24 h (**B**,**C**); Gel electrophoresis of dsGFP at 0 h (**B**) and at 24 h (**C**); Lanes: 1—100 bp ladder; 2—dsGFP with 30% sucrose; 3—dsGFP with NR and 30% sucrose; 4—dsGFP with AO and 30% sucrose; 5—30% sucrose only; 6—30% sucrose and NR; 7—30% sucrose and AO.

The lowest concentrations of NR (0.02%) and AO (0.0025%), for which there were more active aphids with dye in the body after 24 h of exposure, were used to determine if the quality of dsRNA was affected 24 h after it was mixed with 30% sucrose and the dyes. Two replicates each of three treatments of artificial diets each comprising 50 µL of 30% sucrose and 500 ng of a 524 bp-long dsRNA corresponding to the green fluorescent protein (GFP) of *Aequorea victoria*, were made, one with AO at 0.0025%, another with NR at 0.02% and a third with no dye added. The feeding sachets were kept at 23–24 °C for 24 h after which the integrity of the dsRNA before mixing with the dyes and 24 h later was assessed using spectrophotometric analysis and gel electrophoresis. The ratio of absorbance at 260 nm and 280 nm was used as a primary measure of nucleic acid purity: 2.0 is generally regarded as ideal for pure RNA. This ratio for the dsRNA in the three treatments were similar before and after mixing with the dyes thus indicating the purity of dsRNA did not change when mixed with 30% sucrose and the concentrations of AO and NR used during the 24 h period (Figure 3A). On the other hand, the ratios of absorbance at 260 nm and 230 nm (a secondary measure of nucleic acid purity) were slightly lower than the optimal for pure RNA which is between 2.0 and 2.2 (Figure 3A). This decrease is perhaps due to the absorbance of sucrose and/or the dyes at 230 nm. When electrophoresed on a 2% agarose gel at 70 V, a significant proportion of dsRNA from all treatments was intact 24 h after mixing with both AO and NR dyes (Figure 3B,C). Although there appears to be a slight smearing of dsGFP the intensities of the bands, before and after mixing with both AO and NR, were sharp indicating there was insignificant change in the integrity of the dsRNA for the 24 h during the experiment.

### 2.4. Addition of Dyes to Feed Also Attracts Aphids to Artificial Feed

**Figure 4:**
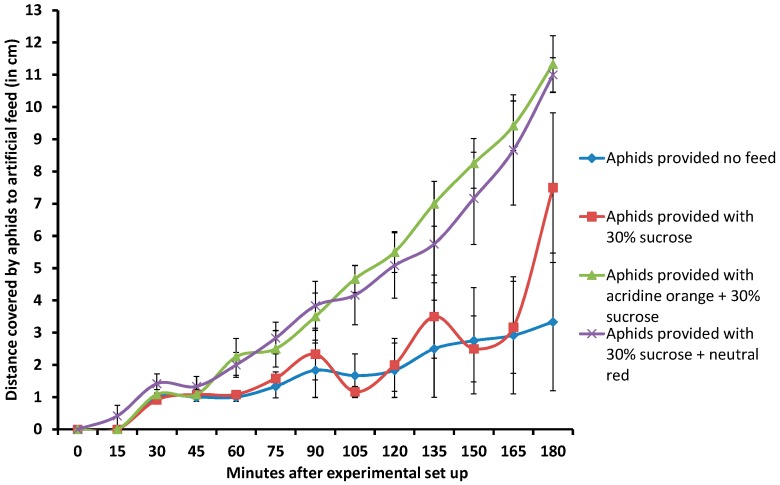
Movement of aphids towards artificial feed supplemented with neutral red and acridine orange dyes. Data is shown as mean± SE of six replicates for each treatment.

In the artificial feeding system used for RNAi studies first described by Mittler and Dadd (1963) and replicated in this study, insects feed ad libitum from solutions between layers of stretched Parafilm M^®^. We tested the hypothesis that in addition to tracing ingestion of artificial feed by the aphids, the dyes by their nature will also attract the insects to the feed. A migration assay to measure movement of nymphs of GPA towards a 100 μL artificial diet containing the dyes NR (0.02%) and AO (0.0025%) mixed with 30% sucrose over a period of three hours was then carried out. Two control experiments were set up with similar stage aphids (2nd–3rd instars); one provided with no feed and the other with 100 μL of 30% sucrose. For each assay, one aphid per channel was placed at one end of a 12-channel isoelectric focusing tray (each channel was 15 cm long, 0.5 cm wide and 1 cm deep) and the distance between aphids and artificial feed at the other end measured at 15 min intervals for 3 h. The assay for each treatment was replicated six times. The average distance between aphids provided with NR-containing feed was shorter than that between aphids provided with AO at each time point observed (Figure 4). Within the third hour, aphids provided with NR and AO had moved closer to the feed; followed by those presented with sucrose alone. Aphids presented with sucrose only diet and those unfed moved back and forth over the 3 h period and this is reflected by the high standard errors of the means (Figure 4). This was probably because they were not attracted by the feed in contrast to aphids provided with a 30% sucrose solution containing these dyes. During the 3-h period, no aphid was observed to have fed, but 21 h later, NR and AO were clearly present in all the aphids provided with these dyes. These observations suggest that incorporating the dyes not only enabled tracing of ingestion of the feed but also attracted the aphids and probably promoted feeding. Several species of aphids are known to have receptors sensitive to light/color wavelengths in the range of 590–595 nm and 425–460 nm with yellow color (580 nm) being the most attractive. Perception of colors in these ranges plays an important role in the behavior of these insects especially in the mechanics of probing, flying and walking. The attraction of the aphids to feeds containing both dyes could therefore be a result of the wavelengths of the dyes. Although the amount of feed ingested was not quantified, it is possible the aphid’s proximity to the feed and appetite for sucrose could then promote feeding.

### 2.5. Tracing Ingestion of dsRNA of Vha-8 by GPAs Allows Efficient Assessment of RNAi Effects

Since the aim of this research was to improve analysis of RNAi after oral delivery of dsRNA to insects, we added the optimised concentration of neutral red, 0.02%, to dsRNA re-suspended in 30% sucrose and fed it to GPA nymphs to determine whether the dye would attract the insects to feed (on dsRNA), and whether feeding could lead to greater down-regulation and RNAi effects in aphids identified as having fed compared to aphids provided with dsRNA without the dye (which may or may not have fed). To do this, 2 µg/µL dsRNA of the vacuolar (H^+^)-ATPase subunit E-like (*vha-8*) gene of GPA (hereafter referred to as *dsMpVha-8*), suspended in 30% sucrose with or without neutral red (0.02%) was fed to two replicates of twelve 2nd to 3rd instars of GPA and RNAi effects analysed. As controls, two replicates of 12 nymphs at similar developmental stages were also fed with dsGFP in 30% sucrose and on 30% sucrose alone, both with and without neutral red and also on water alone.

**Figure 5:**
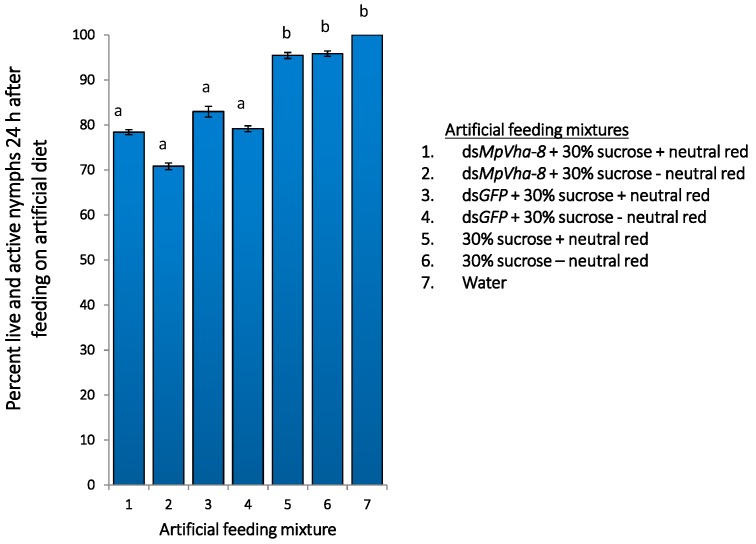
Percentage of active aphids after 24 h of feeding on dsRNA with and without NR. Data is shown as mean ± SE of two replicates with *n* = 12 nymphs per replicate. Same letters denote no significant differences (*p* < 0.05).

For treatments with dye, over 80% of nymphs migrated towards the feed within 30 min of the experimental set-up, compared to nymphs presented with feed without dye, indicating the dye attracted the insects to the feed. On average 11 out of 12 aphids for each treatment with dye had the gut and stylet stained after 24 h. Aphid activity and changes in behaviour were observed after 24 h feeding. There was no significant difference in activity of aphids fed the three control diets without dsRNA (*p* < 0.05, Figure 5). In addition, addition of the dye to the feed did not seem to affect activity of the aphids as the proportion of live and active aphids after 24 h of feeding from similar mixtures with and without the dye were not significantly different (*p* < 0.05) (Figure 5). However, aphid activity was significantly reduced when fed on *dsMpVha-8* mixed with neutral red (78.4% alive and active) and without dye (70.8% alive and active) compared to the number of live and active nymphs from all the three no-dsRNA and dsGFP controls (*p* < 0.05, Figure 5).

**Figure 6:**
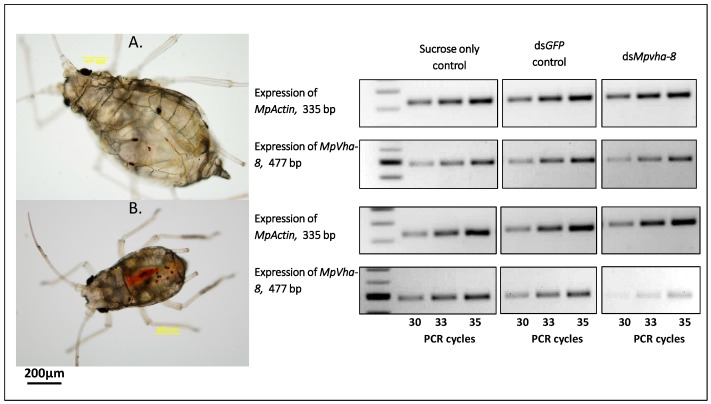
Semi-quantitative PCR analysis of *MpActin* and *MpVha-8* genes. Amplicons of *MpActin* and *MpVha-8* from aphids provided with artificial diets with dsRNA and no-dsRNA controls without dye (**A**); and with dye (**B**).

Transcript abundance of the *vha-8* gene in aphids that fed on target dsRNA was compared to that in aphids fed dsGFP and sucrose only, with and without dye, using semi-quantitative PCR. RNA was extracted from only live NR-stained nymphs after 24 h (for those fed with NR) and all live nymphs fed without dye. Transcript abundance was assessed using band intensities of three replicates of amplicons obtained after the 30th, 33rd and 35th PCR cycles on agarose gels. Using this method, the differences in transcript abundance were clear in all replicates of the PCRs as represented in Figure 6. At each of the three cycles the expression of actin was similar for aphids in all the treatments (Figure 6A,B). Feeding on dsGFP did not seem to affect the *vha-8* target gene expression as the accumulation of *vha-8* transcripts did not differ from that observed for aphids which fed on sucrose alone (Figure 6A,B). However, in aphids that had ingested *dsMpVha-8* with dye there was less transcript accumulation of the target gene compared to that in aphids fed with *dsMpVha-8* without dye (Figure 6B). This shows that there was a more pronounced gene knockdown when NR-stained aphids alone were assessed compared to pooled samples from aphids provided with dsRNA without the dye. In addition, the quality of dsRNA before and after the feeding experiment was compared: since there was no significant difference in the two measurements it can be concluded that during ingestion, aphids did not secrete substances which degraded the dsRNA.

**Figure 7:**
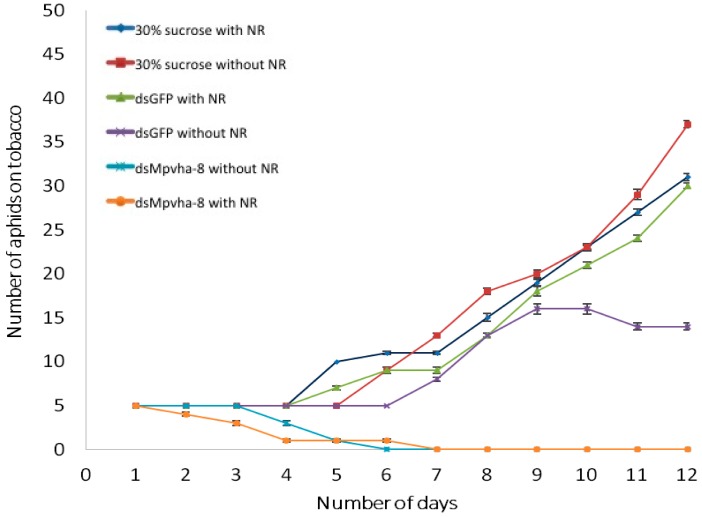
Survival of aphids previously fed artificial diets with and without dsRNA and/or neutral red for 24 h, on tobacco. Data is shown as mean ± SE of five replicates for each treatment.

To determine longer-term effects of ingesting *dsMpVha-8* on aphid survival and reproduction, and whether this effect was more pronounced in aphids with visible evidence of ingesting dsRNA, five live aphids with dye in the stylet or gut after feeding on each of *dsMpVha-8*, *dsGFP*, and sucrose alone (without dsRNA), and five from similar treatments without dye were transferred to tobacco plants, one per plant and their development and reproduction monitored over 12 days. On average nymphs fed 30% sucrose alone reproduced five days after transfer onto tobacco plants while it took six days for aphids fed *dsGFP* to reproduce (Figure 7). In contrast, for aphids fed *dsMpVha-8* (with or without dye) the primary aphid died by the 6th day. For aphids fed with *dsMpVha-8* with dye, a steady decline in aphid numbers was obvious one day after they were transferred onto tobacco plants, where 20% died, and by the third day approximately 20% of the initial aphids remained active on plants. Mortality of aphids fed on *dsMpVha-8* (without dye) was not as dramatic: it took until the 3rd day to observe any death (40% of the total). This result is similar to reported gene silencing effects on several insect species where oral ingestion of different sizes and amounts of dsRNAs of V-ATPase subunits cause gene knockdown, resulting in negative impacts on growth and development, and in some cases mortality in insects such as *D. virgifera virgifera*, *Leptinotarsa decemlineata*, *Drosophila melanogaster*, *Tribolium castaneum*, *A. pisum* and *Manduca sexta and B. tabaci* [7,37,43,44]. The reduced reproductive rate of aphids after ingestion of dsRNA of *vha-8* and the demonstration that feeding on transgenic plants expressing hairpins of the *vha-8* gene has a similar effect on *B. tabaci*, *D. virgifera virgifera* and *M. persicae* support the theory that RNAi has the potential to be developed as a pest control strategy which can contribute to existing pest management strategies to reduce the extensive damage caused by insects to agriculture.

## 3. Materials and Methods

### 3.1. Insect Rearing on Tobacco Plants

The GPA lineage used in this study was obtained from the Plant Pathology Division of the Department of Agriculture and Food, Western Australia. The insects were reared on *Nicotiana tabacum* kept in custom-built mesh cages and/or acrylic cages at 23–24 °C under a 16:8 h light: dark cycle. To understand the longer-term effects of ingesting dsRNA or dye on reproduction, in vitro treated nymphs fed dsRNA were transferred to 2-week old tobacco seedlings grown in 620 mL clear plastic disposable cups filled with soil. Clear plastic lids were placed tightly on the cups immediately after the aphids were transferred. The lids had a small window of 1.5 × 1.5 cm covered with cloth mesh for gas exchange.

### 3.2. In Vitro Artificial Feeding Assays

The artificial feeding mixtures (diet), 30% sucrose with or without dsRNA and/or dye, were fed to nymphs in feeding chambers similar to the one described by Mittler and Dadd (1963). To make a feeding chamber GPA nymphs were first collected carefully from tobacco plants and transferred to a 5 mL clear yellow cap plastic container (Sarstedt, Mawson Lakes, SA, Australia) using a fine paintbrush. The containers, without lids, were then placed tightly in a 24-well plate while feeding sachets were constructed. To make a feeding sachet, about 2 cm^2^ pieces of Parafilm M^®^ (Pechiney Plastic Packaging, Chicago, IL, USA) were cut and cleaned with 100% ethanol and also with RNaseZap (Thermo Fisher Scientific, Scoresby, VIC, Australia). A thin layer of Parafilm M^®^ was stretched carefully and quickly to cover the bottles containing the aphids. The feeding mixture, usually 50 µL, was thus sandwiched between two stretched layers of Parafilm M^®^. Feeding chambers with aphids were kept undisturbed for 24 h at 21–23 °C, after which phenotypic observations were made. Before dis-assembling the feeding chamber, aphids were carefully observed for changes in behaviour compared to controls, and later with a compound and/or a dissecting microscope during which activity of aphids (change in movement or reaction in response to an external stimuli, fine paint brush) was assessed. All data (e.g., numbers of aphids, activity and distances between aphid and feed in a migration assay) were expressed as means with standard errors and mean differences analysed using the Student’s *t*-test with unequal variances.

### 3.3. Identification and Amplification of MpVha-8

**Figure 8:**
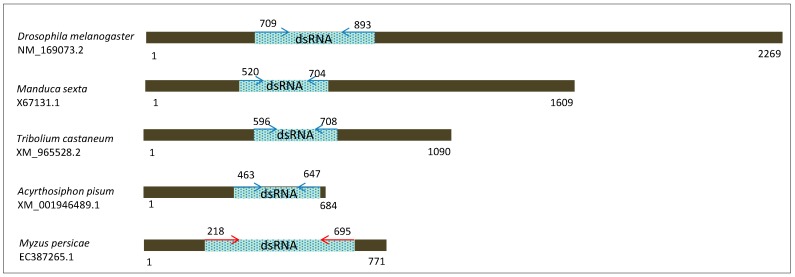
Schematic diagram of the vacuolar proton pump subunit E (V-ATPase subunit E) mRNA sequences depicting the target regions used for dsRNA synthesis in five insect species. Blue arrows with numbers indicate primer positions for a 185 bp-long target sequence amplified from *Drosophila melanogaster*, *Manduca sexta*, *Tribolium castaneum*, *Acyrthosiphon pisum* for RNAi studies (Whyard et al., 2009) and the red arrows with numbers indicate primer positions for a 477 bp-long target sequence from *Myzus persicae* used in the current study.

An expressed sequence tag (EST) of the vacuolar (H^+^)-ATPase subunit E-like, designated *MpVha-8* was used as the target sequence to assess effects of feeding dsRNA (suspended in sucrose solution) with or without dye. The sequence was identified after TBLASTX search of the non-redundant nucleotide insect database of the National Center for Biotechnology Information (NCBI) using *C. elegans vha-8* sequence (C17H12.14, wormbase.org): this resulted in identification of several ESTs of *M. persicae* with e-values less than 1 × 10^−5^. The *M. persicae* EST with Genbank accession number EC387265 (771 nucleotides) with a maximum and total score of 212, 98% coverage and 54% identity was selected for amplification using the primer pair (F: 5′-TCACTCGAGCGTCTGGTCCACAC-3′ and R: 5′-TCAGGTACCTCAGTCCAATCGCGATTC-3′) which amplified a 477 bp fragment from cDNA generated with the High Capacity Transcription kit (Applied Biosystems, Mulgrave, VIC, Australia). The cDNA was synthesised from RNA obtained from mixed life stages of GPA which was extracted as follows: 200 aphids were mechanically lysed with liquid nitrogen into powder, 600 µL of TRIzol LS Reagent (Thermo Fisher Scientific, Scoresby, VIC, Australia) diluted with 200 µL of RNAse-free water was then added and the mixture was vortexed briefly and then incubated at room temperature for 5 min. After centrifugation (12,000× *g*) for 5 min the aqueous phase was then extracted with 200 µL of chloroform and then precipitated with 0.1 volume of 5 M sodium acetate (pH 5.2) and 2 volumes of ice cold 100% ethanol after an overnight incubation at −20 °C. The nucleic acids were then centrifuged at 12,000× *g* at 4 °C, washed by centrifugation at 12,000 *g* with 200 µL of 80% ethanol and the pellet resuspended in 20 µL of RNAse-free water. The RNA was then treated with Qiagen RNAse-free DNase I according to the manufacturer’s protocol (Qiagen Pty Ltd., Malvern East, VIC, Australia) and later retrieved by chloroform extraction and salt precipitation as described above. cDNA was generated using the High Capacity cDNA Reverse Transcription kit (Thermo Fisher Scientific, Scoresby, VIC, Australia) following the manufacturer’s protocol). PCRs were conducted using 300 ng cDNA and the MyTaq DNA Polymerase (Bioline, Eveleigh, NSW, Australia) following the manufacturer’s protocol in a Veriti 96-Well Thermal Cycler (Applied Biosystems) at 95 °C for 3 min followed by 35 cycles of denaturation at 95 °C for 30 s, primer annealing at 60 °C for 30 s, extension at 72 °C for 30 s and a final extension at 72 °C for 7 min. The amplicon was eluted from 1% agarose gels, purified using the Wizard^®^ SV Gel and PCR Clean-Up System, (Promega Corp., Madison, WI, Australia), cloned using the pGEM-T vector (Promega Corp.) and sequenced using an ABI 3730 96 capillary system. The cloned 477 bp EST (*MpVha-8*) was 99% and 96% identical to EC387265 and *A. pisum* EST NM_001162178.2 respectively and was also identical (82% to 99%) to sequences of *D. melanogaster*, *M. sexta* and *T. castaneum* for which RNAi (of the *vha-8* gene) is reported to have a detrimental effect on development (Figure 8) [43].

### 3.4. Semi-Quantitative RT-PCRs

Semi-quantitative RT-PCRs were used to assess gene knockdown of the *vha-8* gene of GPA after 24 h feeding on target dsRNA. Because RNA was extracted from a small number of aphids (1 to 5), the Arcturus PicoPure RNA Isolation Kit (Thermo Fisher Scientific, Scoresby, VIC, Australia) was used followed by an on-column RNase-free DNase I treatment according to the manufacturer’s protocol (Qiagen Pty Ltd., Malvern East, VIC, Australia). For these assessments, 100 ng of RNA for each treatment was used to synthesise cDNA and subsequently 1:10 dilution of the cDNA was used for PCRs. Gene expressions (transcript abundance) of the *vha-8* and actin genes in aphids fed dsRNA of the target gene and those used in controls were compared after 30, 33 and 35 PCR cycles using the same temperature profile for amplifying the *Mpvha-8* gene (see Identification and amplification of *Mpvha-8*). The primers for amplifying the actin gene, MpActin-F: 5′-TCACTCGAGACAGGTCATCACCATCGGAAACGA-3′ and MpActin-R: 5′-TCAGGTACCTCCACATCTGTTGGAAGGTGGACA-3′ were designed based on *M. persicae* EST, EE261235 (NCBI) and amplified a 335 bp fragment. Semi-quantitative PCRs were carried out in triplicates, the PCR products were then pooled for each experiment and the same volumes of PCR product run on 1% agarose gels.

### 3.5. Synthesis of dsRNA Corresponding to MpVha-8 and GFP

Double-stranded RNA corresponding to 477 bp of *MpVha-8* and a 524 bp long dsRNA of the green fluorescent protein (GFP) of *Aequorea victoria*, representing base 47 to 571 of the m*GFP* (M62653) were synthesised using Hi-Scribe T7 In vitro transcription kit (New England Biolabs, Ipswich, MA, USA). DNA template for the synthesis were generated by first ligating the target amplicons to the transcription vector, pDoubler [45] from which restriction enzyme-digested DNA templates with T7 polymerase promoter sequence flanking the 5′ and 3′ ends of the sequences were used for RNA transcription following the manufacturer’s protocol. DsRNA was then treated with DNase I (Qiagen Pty Ltd., Malvern East, VIC, Australia), phase separated with chloroform and precipitated overnight at −20 °C with 0.1 volumes of 3 M ammonium acetate (pH 5.2) and 2 volumes of 100% ethanol. The dsRNA was then washed with 70% ethanol and the pellet resuspended in 20 μL of RNase-free water. The quantity of, and quality of dsRNA was assessed on a 1.5% non-denaturing gel and with the NanoDrop 2000 Spectrophotometer (Thermo Fisher Scientific, Scoresby, VIC, Australia).

## 4. Conclusions

In this report, we examined the suitability of inexpensive vital dyes as an addition to dsRNA mixtures orally delivered to aphids during in vitro RNAi to study gene function. The aim was to trace and select those aphids with the dye in their body, indicating ingestion of the dsRNA mixture, for further assessment of gene knockdown. This work was necessary because for aphids and other insects where oral delivery of dsRNA is the most appropriate method, there is a need for an effective means of identifying those that actually take up dsRNA to allow accurate assessment of gene silencing, since generally in these feeding systems insects feed ad libitum. We have demonstrated that ingestion of four vital dyes out of the eleven dyes tested, neutral red, acridine orange, congo red and phloxin B, can be monitored in the GPA body after ingestion 24 h after the set-up.

We showed that optimum concentrations of both acridine orange and neutral red attracted insects to artificial feed containing dsRNA and ingestion of the feed containing the dyes did not cause any obvious deleterious effects on insect activity, development or reproduction. This attraction could possibly have encouraged the aphids to feed more, resulting in a more pronounced gene silencing, at least for the *MpVha-8* gene studied. It was clear that when only neutral red-stained aphids that had ingested dsRNA mixture with the dye were analysed, there was a more pronounced gene knockdown and effects of silencing on activity and reproduction.

To our knowledge, this is the first study that makes use of inexpensive vital dyes to trace the uptake of dsRNA by GPAs. The vital dyes used here to demonstrate their potential, as internal markers for feeding-based RNAi studies in insects have many advantages: they are inexpensive compared to fluorescently-labeled dyes (e.g., Cy-3^®^ dye) or using fluorescently-labeled dsRNAs, are readily available, and are easy to visualise in many organs including the stylet, salivary glands and alimentary tract of aphids using a dissecting microscope. For example, neutral red stains in aphid bodies are visible to the naked eye. Using these dyes large-scale RNAi screening of target genes is possible, and this will facilitate the accurate identification of suitable target genes which could be used for host induced gene silencing as a strategy to control insect pests, as demonstrated for *vha-8* and other essential genes [46].

## Figures and Tables

**Table 1:** Activity of GPA nymphs after 24 h feeding with eleven dyes.

Dye Tested with 30% Sucrose	Concentration	Number of GPA Active *	Observation of Dye
Fluorescein isothiocyanate	0.5 mg/mL	10	Similar fluorescence as in untreated aphids
	1 mg/mL	10	Similar fluorescence as in untreated aphids
	2 mg/mL	12	Similar fluorescence as in untreated aphids
Fluorescein diacetate	0.0001 *w*/*v*	13	Similar fluorescence as in untreated aphids
	0.001 *w*/*v*	10	Similar fluorescence as in untreated aphids
	0.01 *w*/*v*	9	Similar fluorescence as in untreated aphids
Red food color	0.50%	13	Undetected
Yellow food color	0.50%	14	Undetected
Aqueous acid fuchsin	0.25%	6	Undetected
Aqueous methylene blue	0.50%	9	Undetected
Aqueous fast green	0.50%	9	Undetected
Phloxine B	0.75%	7	Dye detected in one aphid
Congo red	0.50%	13	Dye detected in two aphids
Neutral red	0.50%	15	Dye detected in five aphids
Acridine orange	0.05%	16	Dye detected in six aphids

* The total number of GPA used for testing each dye concentration was 20.

**Table 2:** Average number of GPA that showed the presence of NR and AO after aphids were presented with different concentrations of the dyes.

Treatment	Concentration (%)	Percentage of GPA with Dye
Neutral red with 30% sucrose	0.02	96.7
	0.05	76.7
	0.1	86.7
	0.3	83.3
	0.5	86.7
Neutral red with water	0.02	73.3
Acridine orange with 30% sucrose	0.0025	10
	0.005	90
	0.01	60
	0.03	86.7
	0.05	60
Acridine orange with water	0.0025	40
